# Strengthening Perinatal Services Through Social Care: Outcomes of a Quality Improvement Initiative for a Health Center‐Based Perinatal Care Program

**DOI:** 10.1111/jmwh.70042

**Published:** 2025-10-29

**Authors:** Rebecca L. Emery Tavernier, Peyton Rogers, Mia Shenkman, Aaliyah Moore, Briana Sailor, Veena Channamsetty, Yvette Highsmith, Margaret Flinter

**Affiliations:** ^1^ Weitzman Institute Moses Weitzman Health System Washington District of Columbia; ^2^ University of Minnesota Medical School, Duluth Campus Duluth Minnesota; ^3^ Wesleyan University Middletown Connecticut; ^4^ Community Health Center, Inc Middletown Connecticut

**Keywords:** federally qualified health center, prenatal care, social care, social determinants of health, maternal and child health, health disparities

## Abstract

**Introduction:**

Given the prevalence and consequences of unmet social needs in perinatal populations, there is a critical demand for perinatal care that addresses social needs. To better support health systems in providing comprehensive social and perinatal care services, this quality improvement initiative uses the Donabedian model for care quality to describe the structure, process, and outcomes of embedding an innovative perinatal care program with integrated social care into an established primary care center.

**Process:**

The Improving Maternal Outcomes Now! (IMON) program was designed to address the clinical and health‐related social needs of patients at highest risk of maternal health disparities. The IMON program offers holistic prenatal and postpartum care through the provision of midwifery services, obstetrician support, intensive social needs support, and around‐the‐clock virtual care. Program implementation began in June 2023 at a federally qualified health center.

**Outcomes:**

During the first 18 months of implementation, 102 pregnant patients received prenatal care. Forty‐four percent of patients identified as Hispanic, with more than half (54%) reporting Spanish as their preferred language. Patients were highly engaged with program services. Nearly two‐thirds of IMON patients (65%) initiated prenatal care in their first trimester, and most (91%) were assisted with social needs during or after pregnancy. A majority (88%) enrolled to receive adjunctive virtual care services. Among the 61 patients who gave birth, 77% did so vaginally, whereas the remaining 23% did so via cesarean birth. On average, patients gave birth at 39 weeks’ gestation, with only 5% giving birth preterm and 3% having a newborn that was small for gestational age.

**Discussion:**

Preliminary findings suggest that IMON can be implemented within a safety‐net setting, with high patient engagement and social needs support. Early outcomes show promising maternal and neonatal health indicators.

## INTRODUCTION

Rates of maternal morbidity and mortality in the United States are disproportionality elevated among historically marginalized racial and ethnic groups and communities with low income.^1^, ^2^ Although non‐Hispanic Black and Indigenous birthing people are at highest risk for severe maternal morbidity and mortality,^2^ individuals of certain Hispanic ethnicities also experience undue risk compared with non‐Hispanic White individuals.^3^ Birthing people with lower incomes are similarly more likely to experience perinatal and birth complications than their higher‐income peers, even after accounting for racial and ethnic identity.^1^ Newborns of parents from marginalized racial or ethnic backgrounds and those of low socioeconomic status are likewise at heightened risk for health complications, including premature birth, low birth weight, and infant mortality.^4^, ^5^ These maternal and infant health inequities are largely driven by social and structural determinants rooted in systemic racism and sexism, like unstable housing conditions, economic imbalance, limited insurance coverage, and barriers to quality health care.^6^ Accordingly, initiatives aimed at achieving maternal and infant health equity must address these upstream determinants to be effective.

Despite the prevalence and consequences of unmet social needs in perinatal populations,^7^ efforts to integrate social care into perinatal care delivery are limited, and those that do exist often focus on addressing a single social issue rather than doing so comprehensively.^8^ Social care integration can pose challenges to health systems because it requires screening patients for social needs, incorporating social needs into clinical decision‐making, linking patients to resources, and engaging in organizational partnerships.^9^ These steps can be especially difficult for systems that are underresourced and understaffed like federally qualified health centers (FQHCs). FQHCs provide care at low or no cost and primarily work with patients from underserved communities disproportionately composed of historically marginalized racial and ethnic groups living in poverty.^10^ FQHCs rely on Medicaid reimbursements that are often lower than the cost of providing care and federal funds that have not kept pace with inflation.^11^ These financial burdens, coupled with growing FQHC workforce shortages, are likely to affect care quality and pose challenges for fully integrating social care into primary health services like perinatal care.^12^

**QUICK POINTS**

✦ The Improving Maternal Outcomes Now! (IMON) program is designed for patients at high risk for maternal health disparities.
✦ IMON patients receive comprehensive perinatal care, social needs support, and around‐the‐clock access to virtual care.
✦ Patient outcomes from the IMON program were largely favorable compared with national averages.
✦ Results demonstrate the need for programs and policies that support health centers in developing and implementing integrated perinatal care.

To better support FQHCs and other health systems in integrating social and perinatal care, we describe a quality improvement initiative focused on embedding a comprehensive perinatal care program with intensive social support into a FQHC. This initiative is guided by Donabedian's structure‐process‐outcome framework^13^ for evaluating health care quality and is reported using the Standards for Quality Improvement Reporting Excellence (SQUIRE) guidelines.^14^ Using this framework, we detail the foundational infrastructure established to support the program (structure), the clinical practices and partnerships used to deliver services (process), and the early indicators of patient engagement and health impact (outcomes).

## PROCESS

### Program Overview

According to the Connecticut Department of Health,^15^ the city of New Britain faces persistent inequities that significantly affect community well‐being. Of its 74,000 residents, 63% are people of color, including 44% who identify as Hispanic. Compared with Connecticut rates overall, New Britain has higher poverty (21% vs 10%), lower homeownership (41% vs 66%), lower high school graduation (82% vs 91%), and more uninsured adults (17% vs 10%). Nearly 1 in 5 New Britain residents has limited English proficiency, the median household income is more than $30,000 below the state average, and life expectancy is nearly 4 years shorter. These inequities are reflective of long‐standing barriers to economic opportunity, education, and quality health care and have contributed to rising rates of maternal and infant health disparities.^16^

To better understand the birth experiences of New Britain residents, we conducted a needs assessment with local community members who had given birth in the past 3 years. Interviews were conducted by a Community Health Center, Inc. (CHCI) staff member and followed a semistructured format in which participants were asked to describe their prenatal care experiences and identify areas for improvement. Twelve birthing people were interviewed. On average, participants were 30 years of age. Most participants (75%) had more than one child, with 33% identifying as non‐Hispanic Black and 33% identifying as Hispanic. Rapid qualitative inquiry was applied using matrices to summarize interview transcripts.^17^ Results from these conversations revealed that birthing people in New Britain did not feel listened to by their health care providers and did not feel that their needs were adequately met during their perinatal care, resulting in lower care quality. All participants described wanting more social support.

**Figure 1:**
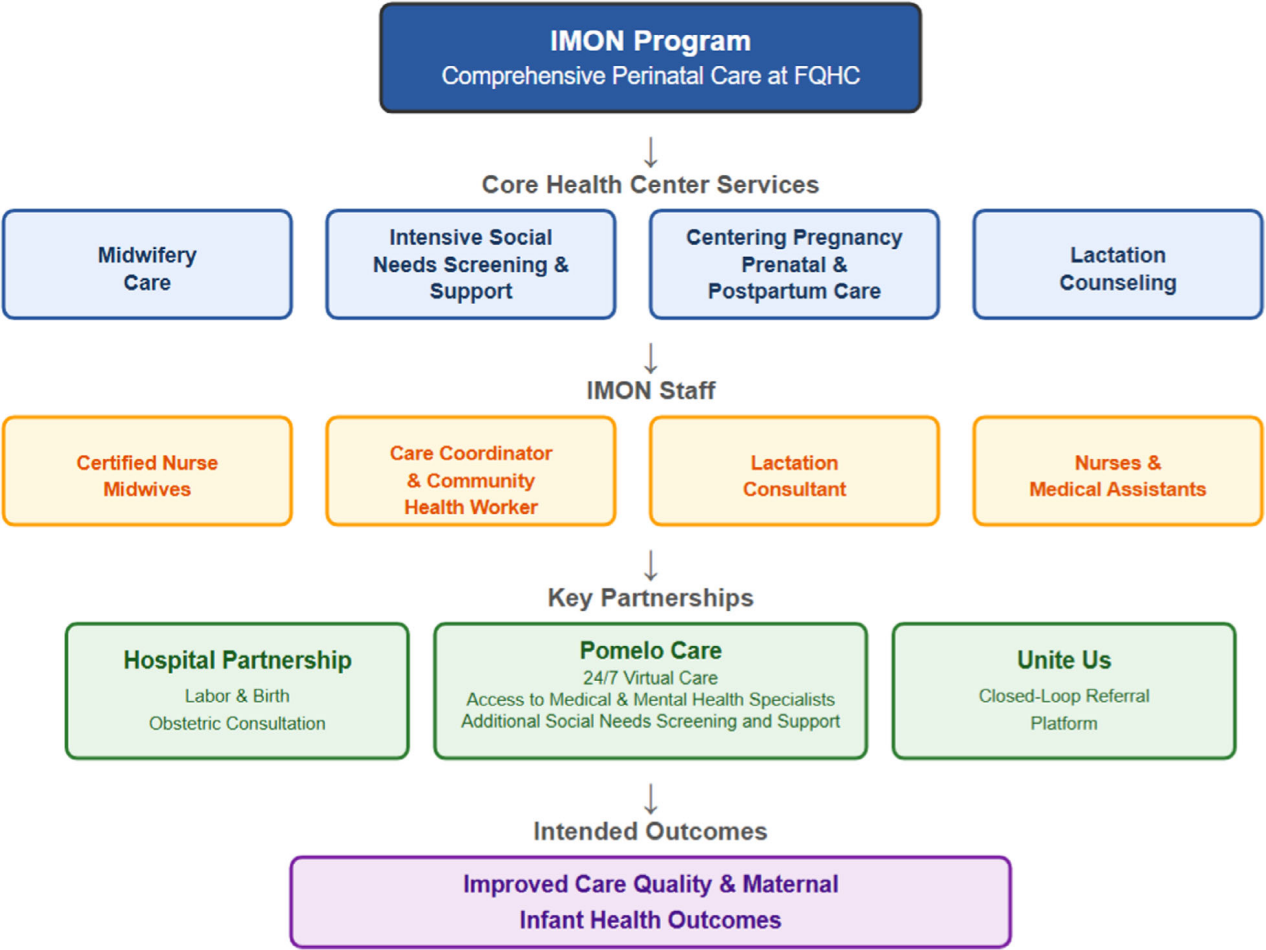
Structure and Goals of the Improving Maternal Outcomes Now! Program Abbreviations: FQHC, federally qualified health center; IMON, Improving Maternal Outcomes Now!.

In response to these community conversations, the Improving Maternal Outcomes Now! program (IMON) was developed. To improve care quality and provide a more culturally concordant birth experience for New Britain residents, IMON is built around a midwifery model of care^18^ and is staffed by a care team that is racially and linguistically aligned with the patient population.^19^ The core team includes 2 certified nurse‐midwives (CNMs), 2 registered nurses, and 2 medical assistants (see Figure 1). Additional support is provided by a care coordinator and community health worker, with extensive experience addressing social needs, and a lactation consultant. All IMON staff are selected for their commitment to holistic, person‐centered care for birthing people and deep familiarity with local community resources. In line with the needs identified from the community, IMON further established partnerships for hospital‐based labor and birth, access to around‐the‐clock adjunctive virtual care, and intensive social needs support to ensure that clinical and health‐related social needs are comprehensively met. This project was determined to be exempt human subjects research by the CHCI Institutional Review Board, and the need for consent was waived.

### Setting

IMON is implemented at CHCI, the largest FQHC in Connecticut. CHCI serves more than 150,000 patients through an extensive network of health centers, school‐based clinics, mobile units, and shelters. Eighty‐nine percent of CHCI patients are at or below 200% of the federal poverty level, 65% are insured through Medicaid, 57% identify as Hispanic, and 32% prefer to receive care in a language other than English.^20^ CHCI of New Britain cares for 20,000 patients annually and is located in an area of concentrated low‐income households, with limited access to other perinatal care sources.

IMON is funded through a Quality Improvement Fund‐Maternal Health award from the Health Resources and Services Administration (HRSA), which provides 2 years of funding to support FQHCs in improving maternal health outcomes. CHCI was awarded funding in June 2023, which continued through August 2025.

A portion of CHCI of New Britain was renovated to support individual and group perinatal care in the same medical home that provides comprehensive medical, dental, and behavioral health care to patients in the community.

### Partnerships

Partnerships were selected for their alignment with community needs and capacity to work with pregnant individuals from diverse backgrounds in a culturally and linguistically responsive way.

#### Hospital Partnership

Although IMON's CNMs are fully qualified to attend births, CHCI prioritized having them available for full‐time practice onsite rather than pursue hospital privileges. Accordingly, we established full labor and birth support from obstetrician‐gynecologists at The Hospital of Central Connecticut (THOCC) by leveraging long‐standing relationships between the leadership at both facilities. The obstetrician‐gynecologists at THOCC provide prenatal consultation, intrapartum care, labor and birth services, and hospital postpartum care to IMON patients. To sustain this partnership and ensure care coordination, we developed a simplified workflow and referral system and an electronic health information exchange. Due to limitations with the health information exchange, health records are currently faxed to THOCC by the IMON clinical team, and real‐time communication between the care teams occurs through a HIPAA‐compliant texting platform. Biweekly case conferences are also held between the partnering obstetrician‐gynecologists at THOCC and the IMON clinical team to discuss the health and social needs of IMON patients and establish care coordination efforts.

#### Virtual Care

As a complement to the perinatal care offered by IMON, we partnered with Pomelo Care,^21^ a virtual care program that provides around‐the‐clock access to a team of perinatal specialists, including obstetrician‐gynecologists, pediatricians, neonatologists, CNMs, dietitians, lactation consultants, and mental health providers, as well as social needs services. We used grant funds to make Pomelo Care available to all interested IMON patients by text, phone, or video using a “per member per month” fee agreement. Under this agreement IMON patients receive unlimited access to Pomelo Care throughout their pregnancy and up to 3 months postpartum at no cost to the individual. Pomelo Care is offered as an adjunctive service to the comprehensive perinatal care provided through IMON. If patients have additional needs or questions beyond what they receive through IMON, they can contact Pomelo Care specialists for additional clinical or social support. The Pomelo Care team meets weekly with the IMON care coordinator and other program staff to facilitate care coordination by reviewing individual care plans and discussing patient needs. Monthly meetings are also held between Pomelo Care and the larger IMON team to review service use.

#### Social Care

To facilitate connections to social services, we partnered with Unite Us, a closed‐loop referral platform that offers comprehensive social needs screening, referral, and care coordination.^22^ Unite Us allows care team members to electronically refer patients to the platform, where they receive care coordination and are connected to community organizations in the Unite Us network. The platform allows the care team to track patient progress to ensure needs are addressed. However, it was quickly learned that Unite Us had sparse community partners in New Britain, which resulted in patient referrals being frequently denied or unmet. After one year of challenges addressing patient needs through Unite Us, the IMON team discontinued using the platform in favor of a free, local 211 service. The IMON care coordinator and community health worker use this 211 service to facilitate patient connections with community resources. For any need not able to be addressed through 211, the care coordinator and community health worker directly connect IMON patients with social services using their own knowledge, resources, and community connections.

### Training

IMON aims to fully implement the CenteringPregnancy model of group prenatal care, a culturally informed approach shown to improve pregnancy and birth outcomes among birthing people of color.^23^, ^24^ The CenteringPregnancy model follows the recommended schedule of 10 group prenatal visits. Although IMON is currently implementing the CenteringPregnancy model, unanticipated staffing challenges during the first 18 months of program implementation delayed staff trainings. During this interim period, patients were encouraged to attend CenteringPregnancy groups virtually through Pomelo Care in addition to the individual prenatal care they received through IMON.

### Clinical Services

IMON began accepting patients in October 2023. Pregnant patients are referred through their established providers at CHCI or via direct access from the community. All patients complete a prenatal intake with a registered nurse, during which time their pregnancy is confirmed, an estimated due date is determined, and perinatal history is obtained. Patients are also provided standard prenatal education and meet with a CNM for further assessment. Patients then meet with the care coordinator to complete social determinants of health screening using a standard screening tool.^25^ Patients with identified social needs are connected to services by the care coordinator. Patients are also introduced to Pomelo Care and complete a consent form to enroll in the service if interested. Following the initial prenatal visit, patients complete their standard prenatal care appointments through IMON with one of the CNMs at the recommended schedule. Patients also complete all recommended prenatal screening and testing (eg, anatomy scan, glucose challenge test) through IMON. Throughout their pregnancy, patients continue meeting with the care coordinator and community health worker to address ongoing or new social needs. They are also encouraged to use Pomelo Care for after‐hours questions and to receive around‐the‐clock access to virtual care in addition to what they receive through IMON.

Patients are educated about the process for giving birth at THOCC and are given the opportunity to tour the facility with a nurse educator and complete a birth plan. When patients are 28 weeks pregnant, IMON staff fax their medical records to the THOCC obstetric team and begin discussing each patient at the biweekly case conferences in preparation for their pending birth. The IMON team continues to provide chart updates and open discussions with the obstetric team at THOCC to ensure seamless care coordination. IMON patients give birth at THOCC, where they also receive hospital postpartum care. After discharge, patients return to CHCI to receive outpatient postpartum care as well as pediatric care for their newborn. They maintain access to Pomelo Care until 3 months postpartum. Throughout their pregnancy and postpartum, patients also continue receiving primary, dental, mental health, and other specialty care at CHCI.

### Measures

#### Patient Demographics and Pregnancy Characteristics

All demographic and pregnancy characteristics were obtained from the electronic health record (EHR). Patient age was determined from patient‐reported date of birth. Patients self‐identified their race and ethnicity; a single race and ethnicity category was then created by combining these responses (eg, a patient who reported their race as Black and their ethnicity as Hispanic was categorized as Hispanic Black). Patients self‐identified their preferred language, and their insurance provider was recorded.

Gestational age at intake was calculated using the estimated due date, and patients were categorized according to what trimester they initiated prenatal care. The number of previous births was used to categorize patients as nulliparous versus parous and pregnancy complications (eg, hypertensive disorders, gestational diabetes) were recorded.

#### Utilization Measures

The total number of prenatal, postpartum, and lactation counseling visits were extracted from the EHR. Social needs were assessed from the EHR using the PRAPARE screen, a standardized tool that identifies the total number of social determinants of health present for any given patient.^25^ The PRAPARE screen is a valid and reliable tool developed by the National Association of Community Health Centers.^26^, ^27^ The PRAPARE screen is widely used in FQHCs and can identify social risks in pregnant patients.^28^, ^29^, ^30^ Engagement in social care was documented by the care coordinator. Social needs applications submitted to the closed‐loop referral platform along with their outcomes were obtained directly from the platform itself. Pomelo Care use was documented monthly by Pomelo Care.

#### Patient Outcome Measures

Birth information, including birth method and newborn birth weight, were extracted from the EHR. Newborns were categorized as small for gestational age if their birth weight was less than 2500 g and large for gestational age if their birth weight was greater than 4000 g. Gestational age at birth was calculated using estimated due date and birth date. Patients who were less than 37 weeks pregnant at the time of birth were considered to have given birth preterm.

## OUTCOMES

### Patient Demographics

Between October 2023 and November 2024, 169 patients completed a prenatal intake through IMON. Sixty‐seven of these patients later discontinued their prenatal care, the majority (n = 55) of whom transferred their prenatal care elsewhere. Twelve were documented as experiencing a miscarriage. Among the 55 patients who transferred care, 15 did so because of a high‐risk pregnancy that required specialist support and 4 moved away from New Britain. The remaining 36 patients did not specify a reason for transferring care. Patients who discontinued care with IMON were more likely to have initiated prenatal care earlier in pregnancy, identified as White or Hispanic, and spoken English compared with those who remained in the program (see Supporting Information: Appendix S1).

Demographic and pregnancy characteristics for the 102 patients who continued to receive care through IMON are displayed in Table 1. IMON patient ages ranged from 16 to 44 years, with patients initiating prenatal care being between 4.0 and 40.4 weeks’ gestation. Fifty patients were identified as having a high‐risk pregnancy, among whom the most commonly occurring complications were age related due to teenage pregnancy or advanced maternal age (n = 27), followed by conditions like maternal obesity (n = 19) and anemia (n = 6).

**Table 1:** Demographic and Pregnancy Characteristics of Improving Maternal Outcomes Now! Patients (N = 102)

Characteristic	Value
**Age, mean (SD), y**	27 (6)
**Gestational age at intake, mean (SD), wk**	14 (8)
**Trimester at intake, n (%)**	
First trimester (0‐13 weeks’ gestation)	66 (65)
Second trimester (14‐27 weeks’ gestation)	24 (24)
Third trimester (28+ weeks’ gestation)	12 (12)
**Parity,** ^a^ **n (%)**	
Nulliparous	39 (39)
Parous	62 (61)
**Race and ethnicity, n (%)**	
Hispanic	45 (44)
Hispanic White	25 (25)
Hispanic Black	4 (4)
Non‐Hispanic White	2 (2)
Non‐Hispanic Black	9 (9)
Multiracial and other race^b^	11 (11)
Unreported race	6 (6)
**Preferred language,** ^a^ **n (%)**	
Spanish	54 (53)
English	34 (34)
Other^c^	13 (13)
**Insurance provider,** ^a^ **n (%)**	
Medicaid	94 (93)
Private insurance	4 (4)
Private insurance with secondary Medicaid coverage	3 (3)

a Data missing for n = 1 patient.

b Other races include Asian (n = 4), American Indian or Alaska Native (n = 2), and multiracial (n = 5).

c Other languages include Arabic (n = 4), Dari (n = 2), French/Haitian Creole (n = 2), Pushto (n = 1), Swahili (n = 2), and Ukrainian (n = 2).

### Service Use

The PRAPARE tool was completed by 80% of IMON patients, all of whom reported at least one social risk, with scores ranging from 1 to 15 with a mean (SD) of 7.6 (3). The care coordinator and community health worker supported 91% of IMON patients with social needs, 38% of whom were assisted with more than one social need. As shown in Table 2, the most common social need addressed involved assistance with obtaining a breast pump. Other needs identified were obtaining diapers, newborn equipment, newborn clothes, and food assistance. Meanwhile, transportation, housing assistance, and immigration services were each addressed in less than 12% of IMON patients requiring social needs assistance. Of the 137 individual needs assisted with, only 10 were able to be submitted to Unite Us, the closed‐loop referral system, which rejected 60% of claims due to a lack of community partners available to address the need.

**Table 2:** Social Needs Addressed Among the 93 Improving Maternal Outcomes Now! Patients Who Received Social Care Services^a^

Social Need	n (%)
Breast pump	82 (88)
Diapers	39 (42)
Newborn clothes	21 (23)
Newborn equipment (eg, car seat, pack ‘n play)	24 (26)
Food	15 (16)
Transportation	11 (12)
Housing	10 (11)
Immigration services	2 (2)

a Categories are not mutually exclusive, meaning that patients could have had multiple social needs addressed.

During the first year of implementation, 90 patients enrolled in Pomelo Care, with 88 engaging in services. The number of Pomelo services accessed by IMON patients each month ranged from 4 to 36, with a mean (SD) of 23.31 (9.36). Forty‐three percent of interactions with Pomelo Care took place outside of normal business hours. The most commonly used service through Pomelo Care was primary, preventive, or urgent care, followed by mental health support, nutrition visits, and social needs and navigation (see Table 3). Additional services included lactation support, doula services, and group care. All Pomelo Care services were in addition to the clinical care and social needs support patients received through IMON.

**Table 3:** Pomelo Care Services Engaged in by the 88 Improving Maternal Outcomes Now! Patients Who Used Virtual Care

Service	n (%)
Primary, preventive, or urgent care	88 (100)
Mental health support	43 (49)
Nutrition visit	39 (44)
Social needs and navigation	33 (38)
Lactation visit	26 (30)
Doula services	13 (15)
Group care^a^	6 (7)

a The mean (SD) of group sessions attended per patient was 7 (3.5).

Sixty‐one IMON patients gave birth during the first 18 months of implementation. Forty‐four percent attended the minimum recommended 10 prenatal care visits, and 30% attended at least one lactation consultation. At the time of this analysis, 42 of these patients were greater than 6 weeks postpartum, with 86% having attended a 6‐week postpartum visit.

### Patient Outcomes

The most common prenatal complications identified among IMON patients were hypertensive disorders (10%) and gestational diabetes (5%). Birth outcomes for the 61 patients who gave birth during the project timeline are displayed in Table 4.

**Table 4:** Birth Outcomes for the 61 Improving Maternal Outcomes Now! Patients Who Gave Birth

Outcome	Value
**Gestational age at birth, mean (SD), wk**	39.4 (1.25)
**Birth term, n (%)**	
Preterm birth	3 (5)
Full term birth	58 (95)
**Birth method, n (%)**	
Vaginal birth	47 (77)
Cesarean birth	14 (23)
**Newborn birth weight, mean (SD), g**	3211.4 (802.9)
**Small for gestational age, n (%)**	2 (3)
**Large for gestational age, n (%)**	9 (15)

## DISCUSSION

The IMON program incorporated a suite of services, including midwifery care, around‐the‐clock virtual care, comprehensive social needs screening and intervention, and hospital partnerships for labor and birth services. During its first 18 months of implementation, IMON served 102 birthing patients, many of whom identified as Hispanic, were insured through Medicaid, and were diagnosed with a high‐risk pregnancy. More than 90% of IMON patients had at least one social need addressed through the program. The initial patient and service outcomes of the IMON program show the potential for integrated perinatal and social care to produce encouraging outcomes in safety‐net settings.

Compared with national rates, IMON patients experienced lower rates of cesarean birth (23% vs 32%),^31^ preterm births (5% vs 10%),^31^ and small for gestational age births (3% vs 11%).^32^ However, the program also saw higher rates of large for gestational age births (15% vs 10%).^33^ Additionally, 36% of patients initiated prenatal care after the first trimester and only 44% attended the recommended 10 prenatal care visits, thereby highlighting opportunities for improvement in early and consistent prenatal care engagement. In response, we are actively identifying barriers and working with the community to develop targeted solutions. Nevertheless, timely prenatal care entry and continued access remains a broader challenge that requires larger social and structural changes.^34^, ^35^

Programs like IMON illustrate the potential of comprehensive prenatal care services with embedded social care to improve maternal and infant health, yet such models remain underused.^8^ Expanding access to these approaches will require broad policy support, including reimbursement policies that enable FQHCs to overcome financial and operational barriers and more effectively serve communities at highest risk for poor perinatal outcomes.^11^, ^12^ IMON's implementation was indeed facilitated by federal funding, strong organizational commitment, and collaboration across both new and existing partners. The program's continued success has supported its sustainability within our health center, where it remains embedded within standard care.

### Limitations

Implementation challenges included limitations with the closed‐loop referral platform and delays in staff training on the CenteringPregnancy model, which required adjustments midstream. Although core IMON services were sustained by the health center after grant funding ended, maintaining partnerships with third‐party partners like Pomelo Care remains financially challenging without ongoing reimbursement mechanisms. These barriers underscore the structural and financial limitations many FQHCs are likely to face in developing and sustaining comprehensive perinatal care models with integrated social care.^11^, ^12^

An additional limitation is that more than half of patients who discontinued care did not report their reason for doing so, limiting our understanding of program attrition. We also did not collect qualitative data for this initial report, which hinders our ability to contextualize our findings using the lived experiences of IMON patients and staff. Finally, we did not assess the degree of overlap between services provided through IMON and Pomelo Care, such as whether the same social needs were addressed by both programs.

## CONCLUSION

The IMON program highlights how investing in FQHCs to develop comprehensive perinatal care programs that target social determinants of health may help improve maternal and infant health. Although future work is needed to better understand whether such models close the gaps in health disparities, continued support and policy innovation are essential to expand and sustain such models in underserved communities.

## CONFLICT OF INTEREST

The authors have no conflicts of interest to disclose.

## Supporting information




**Appendix S1**. Demographic Comparisons of Patients Who Completed a Prenatal Intake and Either Continued or Discontinued Care Through the IMON Program

**Appendix S2**. Standards for Quality Improvement Reporting Excellence (SQUIRE 2.0)
