# Supplementary material for: Strengthening Perinatal Services Through Social Care: Outcomes of a Quality Improvement Initiative for a Health Center‐Based Perinatal Care Program
Source: J Midwifery Womens Health. 2025 Oct 29;71(1):135–42. doi: 10.1111/jmwh.70042 (PMC12914623; doi:10.1111/jmwh.70042)
Supplement: Supplementary file 1 — Appendix S1. Demographic Comparisons of Patients Who Completed a Prenatal Intake and Either Continued or Discontinued Care Through the IMON Program [file JMWH-71-135-s001.docx]

| **Appendix 1. Demographic comparisons of patients who completed a prenatal intake and either continued or discontinued care through the IMON program** | | | |
| --- | --- | --- | --- |
| **Demographic characteristic** | **Continued with IMON (n=102)** | **Discontinued with IMON (n=67)** | *p* |
|  | *Mean ± SD or % (n)* | *Mean ± SD or % (n)* |  |
| **Age, years** | 26.95 ± 6.33 | 26.65 ± 6.64 | 0.77 |
| **Gestational age at intake, weeks^a^** | 13.80 ± 8.29 | 9.91 ± 5.60 | 0.002 |
| **Trimester at intake^a^** |  |  | 0.01 |
| First trimester | 64.7% (66) | 84.2% (48) |  |
| Second trimester | 23.5% (24) | 14.0% (8) |  |
| Third trimester | 11.8% (12) | 1.5% (1) |  |
| **Parity^b^,^c^** |  |  | 0.31 |
| Nulliparous | 38.6% (39) | 49.2% (29) |  |
| Multiparous | 61.4% (62) | 50.8% (30) |  |
| **Race** |  |  | <.001 |
| Hispanic/Latine | 44.1% (45) | 62.7% (42) |  |
| Hispanic White | 24.5% (25) | 3.0% (2) |  |
| Hispanic Black | 3.9% (4) | 0% (0) |  |
| Non-Hispanic White | 2.0%( 2) | 11.9% (8) |  |
| Non-Hispanic Black | 8.8% (9) | 11.9% (8) |  |
| Other Race | 10.8% (11) | 1.5% (1) |  |
| Unreported Race | 5.9% (6) | 9.0% (6) |  |
| **Preferred Language^b^** |  |  | <.001 |
| Spanish | 53.5% (54) | 70.1% (47) |  |
| English | 33.7% (34) | 29.9% (20) |  |
| Other | 12.9% (13) | 0% (0) |  |
| **Insurance Provider^b^** |  |  | 0.02 |
| Medicaid | 92.2% (94) | 85.1% (57) |  |
| Private insurance | 3.9% (4) | 0% (0) |  |
| Private insurance with secondary Medicaid coverage | 2.9% (3) | 10.4% (7) |  |
| Self-pay | 0% (0) | 4.5% (3) |  |
| Note: Independent samples t-tests were conducted for continuous demographics while Fisher's exact tests were conducted for categorical outcomes ^a^Data missing for n=10 patients who discontinued care with IMON  ^b^Data missing for n=1 patient who continued care with IMON ^c^ Data missing for n=8 patients who discontinued care with IMON | | | |
